# Root-exuded sugars as drivers of rhizosphere microbiome assembly

**DOI:** 10.5511/plantbiotechnology.25.0214a

**Published:** 2025-09-25

**Authors:** Niarsi Merry Hemelda, Yoshiteru Noutoshi

**Affiliations:** 1 Department of Biology, Faculty of Mathematics and Natural Sciences, University of Indonesia, Depok 16424, Indonesia; 2 Graduate School of Environmental, Life, Natural Science and Technology, Okayama University, Okayama 700-8530, Japan

**Keywords:** carbon sources, plant-derived sugars, plant-microbe interactions, rhizosphere, root exudate

## Abstract

Sugars in root exudates play a pivotal role in shaping plant-microbe interactions in the rhizosphere, serving as carbon sources and signaling molecules that orchestrate microbial behavior, community structure, and plant resilience. Recent research has shed light on the dynamics of sugar levels in root exudates, the factors that influence their secretion, and the mechanisms by which these sugars drive microbial colonization and community assembly in the rhizosphere. Microbial communities, in turn, contribute to plant physiological changes that enhance growth and stress tolerance. While well-studied sugars such as glucose, sucrose, and fructose are known to promote chemotaxis, motility, and biofilm formation, emerging evidence suggests that less-studied sugars like arabinose and trehalose may also play significant roles in microbial interactions and stress resilience. Key challenges remain, including the accurate measurement of labile sugars that are rapidly metabolized by microbes, and the elucidation of genetic mechanisms underlying rhizosphere metabolic interactions in both host plants and microbes. Addressing these challenges will advance our understanding of sugar-mediated interactions and inform the development of sustainable agricultural innovations.

## Introduction

Sugars play a crucial role in soil ecosystems, serving as an essential energy source for soil organisms. Despite their importance, the concentration of sugars in soil remains low, primarily due to their rapid uptake by soil microbes, which utilize them as readily accessible carbon sources (Gunina and Kuzyakov 2015; Hütsch et al. 2002). The predominant source of sugars in soils originates from plant biomass, particularly in the form of litter. During decomposition, the primary structural constituents of plant biomass—cellulose and hemicellulose—are enzymatically broken down into their monomeric sugar units. However, this decomposition is a time-intensive process that often takes several months to complete (Gunina and Kuzyakov 2015).

Beyond plant litter, roots are another significant source of sugars in soil. Plants allocate approximately 14–18% of their photosynthetically derived carbon to root exudation (Hütsch et al. 2002). Root exudates are chemically diverse mixtures of both primary and secondary metabolites that are actively released into the rhizosphere. Among the primary metabolites, sugars are typically one of the most abundant constituents (Badri and Vivanco 2009; Vives-Peris et al. 2020), although their composition and abundance show considerable variation among different plant species (Hütsch et al. 2002; McLaughlin et al. 2023; Okubo et al. 2016). Consequently, the rhizosphere is considered a niche enriched in carbon sources compared to the bulk soil.

Interestingly, the rhizosphere microbiome is more abundant but less complex than that in bulk soil. Many studies have shown that the rhizosphere has lower microbial α-diversity than the bulk soil (Bulgarelli et al. 2015; Ling et al. 2022; Lopes et al. 2022; Wei et al. 2023). This reduced diversity is attributed to the strong influence of root exudate. This is supported by an investigation by Afzal et al. (2024) showing that bacterial communities in bulk soil can be transformed into rhizosphere-like communities by the addition of a synthetic root exudate cocktail that mimics wheat root exudates. The β-diversity showed different community compositions before and after cocktail exposure, with fast-growing taxa dominating the population only within 24 h. The composition and amounts of root exudates change in response to the physiological status of the plant, which is influenced by developmental stages (Chaparro et al. 2013; Lopes et al. 2022; Zhao et al. 2021), nutrient availability (Carvalhais et al. 2011; Tawaraya et al. 2013), and the presence of environmental microbes (Ankati et al. 2019; Gu et al. 2016). These findings suggest that the rhizosphere microbiome is shaped through the selective recruitment of bulk soil microbes with functional capacities to adapt to the dynamic nature of root exudates (Afzal et al. 2024; Chaparro et al. 2013; Lopes et al. 2022).

Several studies have highlighted crucial traits required for rhizospheric microbes to colonize this niche, including chemotaxis, motility, adherence to root surfaces, and evasion of plant immunity (Bulgarelli et al. 2015; Jacoby et al. 2018). However, the most fundamental requirement for rhizosphere colonization is arguably the ability to utilize root-derived nutrients effectively. This is supported by findings showing that the rhizosphere harbors distinct microbial communities compared to bulk soil, with the former being enriched in dominant taxa such as Bacteroidetes and Proteobacteria. These taxa are copiotrophs, which have rapid growth rates and thrive in nutrient-rich environments. In contrast, bulk soil is dominated by certain phyla such as Acidobacteria, which are oligotrophic bacteria with slower growth rates that adapt to nutrient-poor conditions (Afzal et al. 2024; Ling et al. 2022; López et al. 2023). Although the rhizosphere is rich in carbon sources, these compounds are rapidly consumed by microbes upon release from roots. For example, microbial uptake of glucose occurs within one minute of its addition to the soil (Jones and Murphy 2007). Once internalized, glucose-derived carbon (C glucose) is mineralized to CO_2_ at a rate of 1.1% min^−1^; at this rate, half of the C glucose can be mineralized within one hour. Alternatively, C glucose is incorporated into microbial metabolites, passing through the bacterial biochemical cycle only about 30 min after uptake (Gunina and Kuzyakov 2015). This rapid turnover highlights the intense competition among microbial communities for carbon sources in the rhizosphere. Consequently, copiotrophs have a competitive advantage in such a dynamic environment due to their rapid growth rates and efficient nutrient utilization compared to oligotrophs.

Among the carbon sources available in root exudates, sugars are perhaps the best studied, despite the current emerging focus on lipids as carbon sources (Yang et al. 2024). Sugars are well known for their roles in plant-microbe interactions in the rhizosphere, both as primary carbon sources and as signaling molecules that influence microbial behavior and community composition. As carbon sources, sugars provide the energy required for microbial growth and activity, thereby facilitating the establishment of root-associated microbiomes (Chaparro et al. 2013; Cole et al. 2017; Lopes et al. 2022). In addition to their metabolic role, sugars such as glucose, sucrose, and fructose act as chemoattractants and modulators of microbial traits such as motility, chemotaxis, and biofilm formation, all of which are critical for effective root colonization (Bhattacharjee et al. 2012; Feng et al. 2018; Tian et al. 2021). In addition to serving as carbon sources that support microbial growth, sugars actively shape the rhizosphere microbiome by selectively recruiting beneficial microbes and mediating plant defense mechanisms against pathogens (Jain et al. 2020). This dual functionality as both metabolic substrates and signaling entities, highlights the central role of sugars in orchestrating plant-microbe interactions (Mesny et al. 2023; Yang et al. 2024). Taken together, these multiple roles of sugars underscore their importance in enhancing plant resilience and productivity under diverse environmental conditions.

This review synthesizes current knowledge on root exudate sugar-mediated plant-microbe interactions, with a particular focus on the role of sugars as carbon sources and signaling molecules. It examines key aspects such as the dynamics of sugar levels in root exudates, the factors influencing sugar secretion, and their role in microbial community assembly and colonization patterns. The review also addresses key methodological challenges, such as accurate quantification of labile sugars and bridging the gap between genomic and metabolomic data to understand the genetic basis of sugar diversity. By integrating recent findings, we illuminate the regulatory mechanisms governing sugar-mediated interactions and their applications for sustainable agricultural practices.

## Dynamics of sugar level in root exudates

### How much sugar is in root exudates?

Studies of root exudates have continually evolved to identify effective methods for collecting exudates under conditions that closely mimic the natural environment. Root exudates and their constituents are species-specific (McLaughlin et al. 2023), highly influenced by plant physiological status (Chaparro et al. 2013; Lopes et al. 2022; Zhao et al. 2021), and environmental factors (Carvalhais et al. 2011; Tawaraya et al. 2013), including the presence of surrounding microbes (Ankati et al. 2019; Gu et al. 2016). Consequently, the choice of root exudate sampling strategy has a significant impact on the observed amount and composition of exudates, as reviewed by Oburger and Jones (2018). For instance, hydroponic systems and soil systems have distinct advantages and limitations.

Soil systems better mimic natural conditions, but they introduce several challenges: root physical damage during harvesting and washing, and reduced exudate concentrations from soil matrix interactions (Oburger and Jones 2018; Williams et al. 2021). Soil systems also raise concerns about sterility, as soils often harbor fungal spores and nematode cysts that can germinate during the culture period. Soil microbes rapidly metabolize available carbon sources like glucose, citrate, and acetate, often making these compounds undetectable (Kuijken et al. 2015). In contrast, hydroponic systems circumvent these limitations by enabling controlled environments and safe root harvesting (Oburger and Jones 2018; Williams et al. 2021). These systems can be maintained aseptically for up to 30 days and can be applied to larger plants, such as wheat and barley (Kawasaki et al. 2018). However, these hydroponic systems also have limitations. Plants grown hydroponically tend to develop a different root morphology compared to soil due to the lack of mechanical impedance, which ultimately affects root exudation (Groleau-Renaud et al. 1998). Another limitation is the ecological relevance. In soil environments, nutrients released by roots diffuse into the bulk soil, creating radial gradients of exudates. These gradients result in diverse microenvironments that influence microbial distribution and activity (Kuzyakov and Razavi 2019; Liu et al. 2022). The rate of exudate diffusion is influenced by factors such as soil water content and sorption of exudates to the soil matrix (Kuzyakov and Razavi 2019), which is not easily replicated in hydroponic systems, where the diffusion rate is generally faster.

Given these challenges, there may be no definitive answer to the question of how much sugar is present in root exudates. Nevertheless, several studies have reported specific sugar profiles under different experimental conditions. These studies illustrate how sugar concentrations in root exudates vary widely depending on plant species, developmental stage, experimental setup, and exudation period. For example, *Juncus effusus* and *Philodendron cordatum* grown in Hoagland’s nutrient solution with sterile tezontle volcanic rock within constructed wetland-microbial fuel cell reactors secreted root exudates containing 319 µg l^−1^ and 246 µg l^−1^ glucose, 297 µg l^−1^ and 339 µg l^−1^ galactose, 381 µg l^−1^ and 330 µg l^−1^ sucrose, and 266 µg l^−1^ and 202 µg l^−1^ fructose, respectively (Guadarrama-Pérez et al. 2024). In another study, root exudates of two barley cultivars grown in the reproductive stage under non-sterile conditions in sand-filled containers showed sucrose, fructose, and glucose concentrations ranging from 0.41–3.15 mg g^−1^ root dry weight (DW), 2.11–3.01 mg g^−1^ root DW, and 2.47–3.14 mg g^−1^ root DW, respectively, for Bambina cultivar, and 0.06–1.88 mg g^−1^ root DW, 2.11–2.48 mg g^−1^ root DW, and 1.75–2.47 mg g^−1^ root DW, respectively, for Golden Promise cultivar (Calvo et al. 2019). Similarly, root exudates of a 1-year-old *Fraxinus mandshurica* sapling grown hydroponically under non-sterile conditions contained 2.59 µg ml^−1^ fucose, 51.08 µg ml^−1^ arabinose, 9.91 µg ml^−1^ galactose, 136.93 µg ml^−1^ glucose, 11.27 µg ml^−1^ xylose, 8.72 µg ml^−1^ mannose, 12.59 µg ml^−1^ fructose, and 12.05 µg ml^−1^ glucuronic acid within 12 h after harvesting. Notably, sugar concentration and diversity decreased significantly as the exudation period was extended beyond 12 h, likely due to reabsorption by the roots, especially glucose, which decreased from 136.93 µg ml^−1^ (12 h) to 4.98 μg ml^−1^ (24 h) (Li et al. 2019). Furthermore, 5-day-old *Arabidopsis thaliana* seedlings grown hydroponically secrete approximately 41 mg l^−1^ sucrose, 8 mg l^−1^ glucose, and 10.5 mg l^−1^ fructose over a 24-h exudation period (Song et al. 2022). Together, these studies demonstrate that sugar concentrations in root exudates fluctuate markedly depending on plant species, developmental stage, experimental setup, and exudation period, highlighting both the dynamic nature of sugar secretion in root exudates and the critical influence of methodological approaches.

### Factors affecting sugar secretion

#### Plant-specific traits

Plant genetics fundamentally shape root exudate composition and sugar profiles, as demonstrated by distinct exudate patterns among species and cultivars (Carrell et al. 2023; Hütsch et al. 2002; Lopes et al. 2022; McLaughlin et al. 2023; Williams et al. 2021). Yet connecting these metabolic signatures to specific genomic features remains challenging, demanding rigorous analysis of candidate genes.

A notable example of genetic control of root exudate composition is provided by Mönchgesang et al. (2016), who reported the absence of a phenolic compound, cyclic didehydro-di(coumaroyl)spermidine sulfate, in several accessions of *A. thaliana* due to a mutation causing a premature stop codon in the gene encoding spermidine dicoumaroyl transferase (SCT). While such studies establish the genetic basis of specific metabolite exudation, investigations focusing on the sugar profiles in root exudates and their genetic regulation are scarce. Nevertheless, promising progress has been made. Subrahmaniam et al. (2025) identified 26 genes associated with 12 compounds, including four carbohydrate-related compounds, through genome-wide association studies. These were associated with genes involved in nutrient transport (*MIR399A*, *ABC4*), cell wall polysaccharide metabolism (*GXM3*), and phenolic compound modulation (*BGLU31*).

Species-specific differences in sugar exudation further illustrate the regulatory complexity. Metabolites such as sucrose, palatinose, trehalose, and glyceraldehyde are consistently found in both roots and root exudates of *A. thaliana*. However, these compounds are restricted to the roots of *Brachypodium distachyon* and *Medicago truncatula* and are absent from their exudates (McLaughlin et al. 2023). This discrepancy suggests differential regulation of sugar exudation among plant species, likely influenced by the distribution of sugar transporters such as SWEETs, which facilitate the diffusion of sugars into the rhizosphere (Loo et al. 2024). Variations in the number of *SWEET* genes, resulting from evolutionary events such as gene duplication, may contribute to the observed differences in sugar transport and exudation (Doidy et al. 2019; Li, Si et al. 2018). Thus, the study of sugar transporters offers a promising approach to unraveling the genetic and metabolic mechanisms underlying sugar exudation.

Developmental stage also significantly affects sugar exudation. In *A. thaliana*, higher levels of sucrose are exuded during early developmental stages, with a decrease observed during later reproductive stages. This pattern is consistent with the expression of several sugar transporters, including AtSUC3, AtINT2, AtINT3, and AtpGlcT (Chaparro et al. 2013). However, contrasting results have been reported in maize, where sugars and their derivatives show higher abundance during reproductive stages (Santangeli et al. 2024). This discrepancy may be due to methodological differences—the study by Chaparro et al. lacked normalization for root biomass—and divergent experimental conditions. While Chaparro et al. used sterile systems, field-based approach by Santangeli et al. included naturally occurring microbes, which likely affected sugar transporter accumulation and subsequent exudation patterns (Loo et al. 2024).

#### Environmental factors

Sugar exudation in plants is strongly influenced by environmental factors, including abiotic conditions such as CO_2_ levels, soil moisture, and nutrient availability, as well as biotic interactions with microorganisms. Elevated CO_2_ levels induce species-specific responses in sugar exudation. In barley, CO_2_ enrichment decreases the exudation of sugars such as sucrose, glucose, and fructose (Calvo et al. 2019), whereas cucumber (Li, Dong et al. 2018) and white lupin (O’Sullivan et al. 2021) exhibit increased sugar exudation. Of the sugars analyzed, glucose and fructose tend to exhibit greater changes (either increases or decreases) in exudation than sucrose (Calvo et al. 2019; Li, Dong et al. 2018). Soil moisture similarly affects sugar exudation patterns. Under moderate water deficit, barley significantly increases sucrose exudation, while glucose and fructose exudation are slightly reduced (Calvo et al. 2019). In soybean and sunflower, drought stress induces root exudation with higher concentrations of sucrose, fructose, glucose, pinitol, and *myo*-inositol (Canarini et al. 2016).

Nutrient availability also has a profound impact on sugar exudation. Under nitrogen (N) deficiency, exudation of glucose, fructose, and sucrose decreases despite their increased concentrations inside the roots (Li, Dong et al. 2018). Unique exudates such as maltose are also detected under N-limited conditions (Carvalhais et al. 2011). Iron (Fe) deficiency promotes the exudation of sucrose, raffinose, galactose, galactinol, and fucose in strawberry (Valentinuzzi et al. 2015) and glucose and ribitol in maize (Carvalhais et al. 2011), which likely supports microbial recruitment to alleviate Fe deficiency. Phosphorus (P) deficiency induces marked shifts in sugar exudation. Sugars and sugar alcohols such as fructose, glucose, arabinose, inositol, erythritol, and ribitol in maize (Carvalhais et al. 2011) and raffinose, galactose, galactinol, and galactaric acid in strawberry (Valentinuzzi et al. 2015) are highly exuded by phosphorus-deficient plants. This enhanced exudation is part of a broader physiological response to phosphorus starvation, where plants prioritize carbon allocation to the roots by reducing invertase and sucrose synthase activity in sink organs, thereby promoting sucrose transport to the roots and its subsequent hydrolysis to hexoses. Interestingly, both Carvalhais et al. (2011) and Valentinuzzi et al. (2015) did not report an increase in sucrose exudation despite the increased allocation of sucrose to roots under P-deficient conditions. This could be due to the hydrolysis of sucrose to its hexoses in roots, as reported by Xiao et al. (2024), who found an elevated hexose/sucrose ratio in roots under P-limiting conditions.

The presence of microbes can influence root exudates, including sugars. However, direct studies of how specific microbes or microbial communities modulate sugar exudation from plant roots are limited. Instead, many studies rely on indirect approaches, such as analysis of sugar content in root extracts (Sun et al. 2022). Alternatively, research often focuses on the activity of sugar transporter genes, such as STPs or SWEETs, in response to symbiotic, beneficial (Desrut et al. 2020, 2021; Manck-Götzenberger and Requena 2016), or pathogenic microbes (Chen et al. 2015; Lemonnier et al. 2014; Yamada et al. 2016). While these methods provide valuable insights into sugar metabolism and its potential impact on exudation, they do not directly measure the sugar content of root exudates. It is important to note that microbially influenced changes in sugar transporters do not always result in altered sugar exudation to the rhizosphere (Breia et al. 2021). In many cases, these changes are aimed at providing sugars to symbiotic microbes to support mutualistic interactions (Manck-Götzenberger and Requena 2016). Conversely, in interactions with pathogenic microbes, transporter activity is often manipulated to direct sugars to the site of infection within plant tissues, thereby increasing microbial access (Hu et al. 2014). Alternatively, plants may modify transporter activity as a defense mechanism to restrict the supply of apoplastic sugars, thereby limiting pathogen proliferation in infected areas (Lemonnier et al. 2014; Yamada et al. 2016).

Despite the predominance of indirect approaches, some studies directly evaluate sugar exudation using root exudates. For example, Chen et al. (2015) demonstrated that *A. thaliana* modulates SWEET2, a glucose transporter localized on the tonoplast of root epidermal cells, during *Pythium* infection. SWEET2 activity facilitates glucose sequestration in the vacuole, thereby reducing the availability of glucose for secretion into the rhizosphere, as evidenced by the decrease in the percentage of exported [^14^C] glucose-derived molecules exported to the rhizosphere, hence limiting the carbon resources accessible to the pathogen. Hoang et al. (2022) demonstrated increased glucose exudation in soybeans under drought stress in the presence of the arbuscular mycorrhizal fungus (*Glomus mosseae*). This was visualized using in situ glucose exudation imaging, which detected glucose release as magenta signals on membranes treated with a glucose-reactive solution. However, the enhanced glucose exudation observed in the study may result from both direct secretion by plant roots and transport via mycorrhizal hyphae. In mature roots, where the Casparian strip restricts the facilitated diffusion of sugars into the rhizosphere, colonization by arbuscular mycorrhizal fungi (AMF) provides an alternative pathway for sugar exudation. Through their hyphae, AMF can transport sugars from root cells to the rhizosphere, bypassing the physical barrier of the Casparian strip, as reported by Kaiser et al. (2015). Similarly, Luo et al. (2022) investigated the effects of *Alternaria panax* foliar infection on *Panax notoginseng* and observed altered root exudate composition, including increased levels of certain sugars (ribose and D-mannose). These sugars were found to recruit and enrich beneficial microbes, such as *Ilyonectria destructans* and *Trichoderma atroviride*, which contribute to the suppression of *A. panax*.

## Sugars influence microbial community dynamics

Root exudates are key drivers of microbial community assembly in the rhizosphere, shaping the composition and activity of the root microbiome. The root microbiome is highly dynamic, with plants actively modulating the composition and quantity of root exudates in response to developmental stages and environmental conditions (Canarini et al. 2019; Chaparro et al. 2013; Lareen et al. 2016; Trivedi et al. 2020). Moreover, different plant genotypes harbor different microbial communities, suggesting that the composition of root exudates driven by plant genotypes may underlie microbial selection in the rhizosphere (Lopes et al. 2022; Pacheco-Moreno et al. 2024; Song et al. 2022). Consequently, the characteristics of root microbiomes are closely linked to the adaptation to the root exudate profile.

Among the various compounds in root exudates, carbohydrates, particularly sugars, play a critical role as carbon sources for microbial growth and colonization. Accumulating evidence highlights the importance of efficient sugar utilization for successful microbial colonization. For example, in *Pseudomonas simiae*, 24 genes involved in the metabolism of specific sugars such as galactose, galacturonate, and glucose were found to be critical for colonization of *A. thaliana* roots (Cole et al. 2017). Similarly, root-associated microbes in sugarcane show significant traits for using sugars, particularly D-galacturonic acid, as a primary carbon source (Lopes et al. 2016). In maize, the composition of root exudate sugars changes dynamically across developmental stages, with sucrose shaping rhizobacterial communities during early growth, while trehalose becomes more prominent in later stages (Lopes et al. 2022). Furthermore, Mesny et al. (2023) revealed that genes involved in carbohydrate metabolism are under positive selection in the *A. thaliana* root microbiome, suggesting that carbohydrate metabolism drives the co-evolution of *A. thaliana* and its associated microbiota. Of course, other factors, such as motility and the ability to evade or modulate plant immunity, further shape the root microbiome (Cole et al. 2017; Lopes et al. 2016; Mesny et al. 2023).

Because catabolic metabolism is an essential trait for rhizosphere colonization, root-associated microbes, particularly bacteria, that actively consume plant-derived carbon, predominate in the rhizosphere. These bacteria are equipped with more genes associated with carbon metabolism and ATP-binding cassette systems associated with sugar uptake, allowing for higher metabolic potential to utilize carbon molecules and rapid growth rates (Fan et al. 2022). Such bacteria, often referred to as copiotrophs (Koch 2001), include taxa like Proteobacteria, Actinobacteria, and Bacteroidetes. Metagenomic analysis by López et al. (2023) revealed that the fast growth potential of these copiotrophs is closely linked to proteins involved in sugar metabolism, such as glycosidases (e.g., beta-galactosidase, α-L-arabinofuranosidase), Na+/melibiose symporters, and fructose/tagatose bisphosphate aldolases. In contrast, oligotrophs such as Acidobacteria, which are adapted to nutrient-poor environments and exhibit slower growth rates, show enrichment in the metabolism of terpenoids, polyketides, and amino acids (López et al. 2023). Although oligotrophs are more commonly enriched in bulk soil than in the rhizosphere, some oligotrophic taxa also colonize the rhizosphere. Their distinct metabolic traits, combined with the heterogeneous distribution of root exudates along the root axis (Loo et al. 2024), suggest that oligotrophs may preferentially colonize specific root regions. Wei et al. (2021) demonstrated that root microbial communities differ spatially, with copiotrophic taxa enriched at the root tip and oligotrophic taxa more abundant at the root base, where alpha diversity is also higher. This spatial differentiation underscores the complexity of root-associated microbial community assembly and highlights the need for further research to clarify whether the observed copiotroph-oligotroph distribution along the root axis is primarily driven by the local availability of specific carbon nutrients or by other ecological factors shaping these dynamic microbial interactions.

## Sugars as carbon sources for microbes

Sugars are critical carbon sources that drive microbial assembly and activity in the rhizosphere. Microorganisms differ in their ability to metabolize various carbon compounds (Table 1). For example, *Bacillus cereus*, *B. anthracis*, and *Staphylococcus aureus* can metabolize 62, 60, and 64 carbon sources, respectively. While 20 of these are shared among species, others remain unique to each, such as 31 carbon sources specific to *B. cereus*, 15 to *B. anthracis*, and 20 to *S. aureus* (Chang et al. 2021). Despite their broad metabolic capabilities, microbes show preferences for specific carbon types. Some bacteria, such as Flavobacteriales, prefer glycolytic (i.e., sugar) over gluconeogenic (i.e., amino and organic acid) carbon sources, while some others, such as Pseudomonadales, exhibit the opposite preference (Gralka et al. 2023; Hemelda et al. 2024; Moreno and Rojo 2024). When two carbon sources are available, their utilization can occur sequentially or simultaneously, depending on the metabolic pathways involved. When two carbon sources enter the same metabolic pathway (e.g., glucose and fructose both enter glycolysis), they are typically used sequentially. However, if the sources are metabolized by different pathways (e.g., glucose and succinate), simultaneous utilization becomes possible. In that case, both can be used simultaneously because the gluconeogenic source (succinate) bypasses the glycolysis pathway and directly supplies downstream precursor pools in the TCA cycle (Wang et al. 2019). This simultaneous use often results in higher growth rates than using a single carbon source alone (Wang et al. 2019). For instance, the addition of organic acids alongside sucrose significantly increases the growth of *Allorhizobium vitis*, including biocontrol and pathogenic strains of crown gall disease, beyond that achieved with sucrose alone (Hemelda et al. 2024; Ishii et al. 2024; Saito et al. 2018).

**Table 1:** Table 1. Role of plant-derived sugars in plant-microbe interaction.

Role	Sugar	Interaction	Mechanism(s)	Reference
Carbon sources	Glucose	*Laccaria bicolor* and poplar	Host-derived sucrose is transported to the root and hydrolyzed into glucose and fructose by plant invertases in the root-ectomycorrhiza interface. Then, glucose is taken up by fungal monosaccharide transporters and converted to fungal-specific storage carbohydrate, trehalose.	Deveau et al. (2008), López et al. (2008)
	Arabinose, Galactose	*Rhizobium leguminosarum* and pea root mucilage	*R. leguminosarum* is able to grow on purified mucilage, mostly contains arabinose and galactose as glycosidic linkages, potentially due to the glucanase activity of this bacterium.	Knee et al. (2001)
	*myo*-inositol	*Streptomyces* sp., *Pantoea* sp. and *A. thaliana*	*myo*-inositol serves as sole carbon sources for *Streptomyces* sp. CL18 and *Pantoea* sp. R4.	O’Banion et al. (2023)
Plant immunity and pathogenicity	Fructose	*A. thaliana* and bacterial communities	Impaired JA signaling in *MEDIATOR25* mutant (*med25*) results in higher fructose levels, correlating with increased populations of *Paenibacillus* spp., *Lysinibacillus* sp., and *Bacillus* sp. in the rhizosphere.	Carvalhais et al. (2015)
	Arabinose	*Ralstonia pseudosolanacearum* and tomato root exudate components	L-arabinose inhibits population of *R. pseudosolanacearum* and reduced disease severity. The mechanism may involve in restriction of proliferation by SA- and ethylene-dependent defense genes that are induced by L-arabinose.	Fu et al. (2020)
	Glucose, Sucrose	*Xanthomonas campestris* and *A. thaliana*	Sucrose and glucose uptake by *X. campestris* stimulate RpfF and RpfC, DSF synthase and sensor, for DSF signals which lead to the enhanced pathogenicity in *A. thaliana*.	Zhang et al. (2019)
Inducible signals	Sucrose	*Trichoderma atroviride* and tomato	Among the investigated sugars, sucrose is the only sugar that is not detected in preinoculation but detected only during root colonization, which indicate *T. atroviride* modulation in sucrose exudation. Even so, the fungal growth in sucrose is not different than in other sugars (i.e., xylose, glucose, *myo*-inositol, fructose).	Macías-Rodríguez et al. (2018)
		*Trichoderma virens* and maize	*T. virens* produce invertase (TvInv) that hydrolyze sucrose in fungal cells, independent of plant invertases. This TvInv activity enhances plant photosynthesis thus stimulate plant to allocate more sucrose to the roots.	Vargas et al. (2009)
Root colonization	Raffinose	*Fusarium oxysporum* and cucumber	Enrichment of raffinose in root exudates is positively correlated to colonization of *F. oxysporum* in cucumber root, yet the mechanisms are unknown. However, cucumber showed to limit raffinose exudation upon *F. oxysporum* inoculation.	Liu et al. (2017)
	*myo*-inositol	*Pantoea* sp. and *A. thaliana*	Plant-derived *myo*-inositol increased *Pantoea* sp. R4 colonization in *A. thaliana* roots. This is mediated by the released of *myo*-inositol by plant inositol transporter INT1 and PMT5, but not the bacterial inositol metabolism by *myo*-inositol dehydrogenase IolG.	O’Banion et al. (2023)
	Sucrose	*Trichoderma virens* and maize	Sucrose preventing overpopulation of *T. virens* in maize root surface, likely mediated by CCR system induced by glucose from sucrose hydrolysis which represses activity of many glucanases and chitinase activity.	Vargas et al. (2009)
Chemotaxis	Glucose	*Rhizobioum leguminosarum* bv. *trifolii* SN10 and rice	Glucose in both seed and root exudates of rice acts as chemoattractant that induce chemotaxis of *R. leguminosarum* bv. *trifolii* important for its colonization on the root surface of rice.	Bhattacharjee et al. (2012)
	Maltose, Fructose, Dulcitol	*Bacillus amyloliquefaciens* SQR9 and cucumber	Maltose, fructose, and dulcitol are chemoattractant to SQR9 that can be recognized by methyl-accepting chemotaxis protein (MCP) TlpB.	Feng et al. (2018)
	Mannose, Fucose, Ribose, Ribitol	*Bacillus amyloliquefaciens* SQR9 and various chemical ligands	Mannose, fucose, ribose, ribitol are chemoattract ligand that can bind directly to McpA chemoreceptor in SQR9. Entire McpA ligand binding domain (McpA-LBD) is needed for interaction with fucose, ribose, and ribitol, while only the distal part of McpA-LBD is needed for mannose binding.	Feng et al. (2022)
	Galactose	*Bacillus velezensis* SQR9 and cucumber	Galactose serves as a strong chemoattractant that perceived by McpA indirectly probably through a galactose-binding protein, induces biofilm formation dependent of McpA, and potentially serves as inducible signals secreted by root in presence of SQR9.	Liu et al. (2020)
Biofilm	*myo*-inositol	*Streptomyces* sp., *Pantoea* sp. and *A. thaliana*	*myo*-inositol alters bacterial colony morphology in which increase the extracellular matrix component qualitatively, indicating a potential induction of biofilm.	O’Banion et al. (2023)
	Sucrose	*Bacillus subtilis* and *A. thaliana*	Sucrose induces swarming motility and biofilm formation mediated by increased surfactin. Production induced by hydrolysis of sucrose to levan by SacB, resulted in increased colonization in *A. thaliana* roots.	Tian et al. (2021)

**Figure 1:**
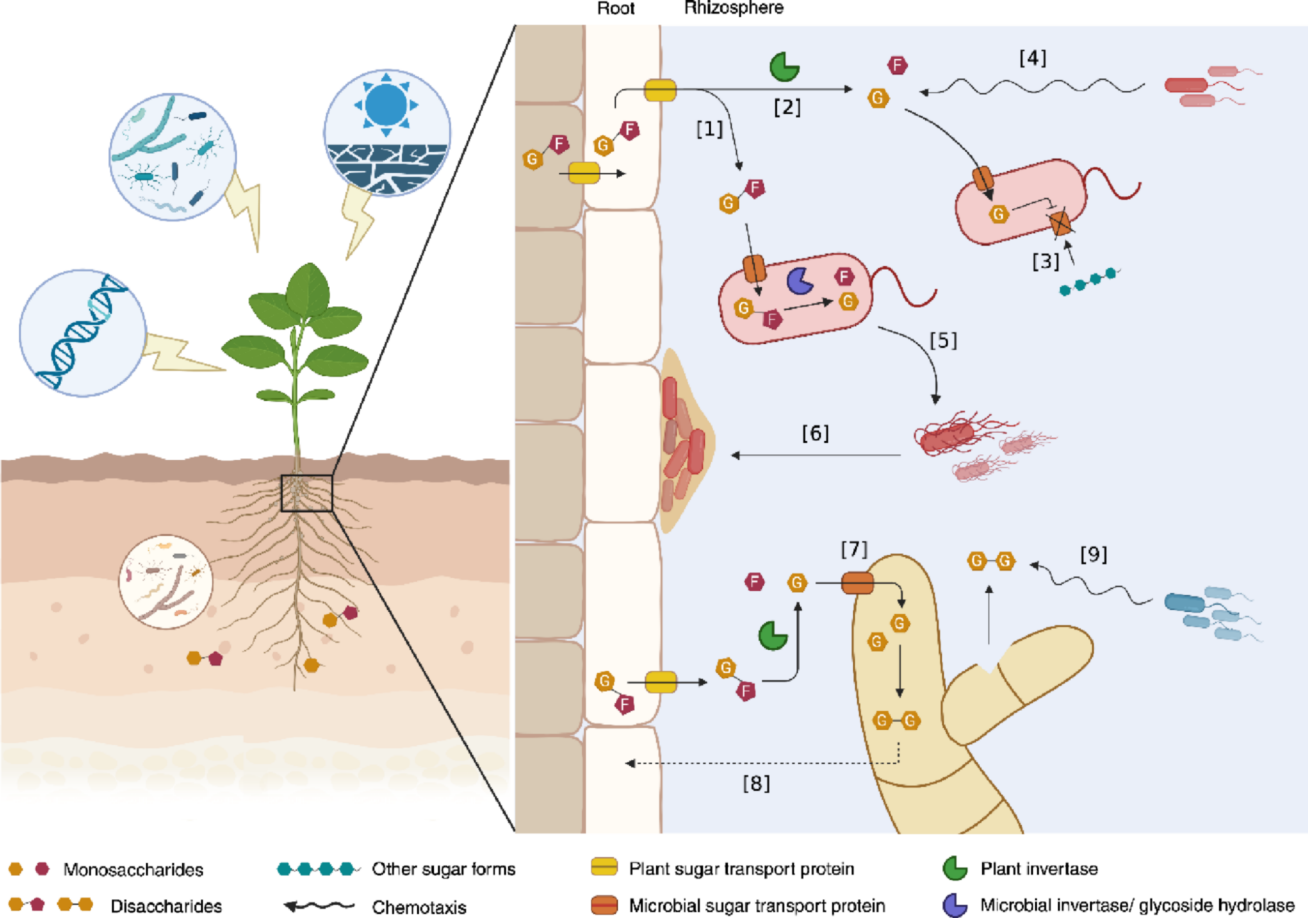
Figure 1. Dynamics of sugars in root exudates and their role in plant-microbe interactions. Sugars exuded from plant roots are influenced by both the plant genetic and environmental factors that affect the physiological responses. This diagram illustrates how sugars function in microbial interactions in the rhizosphere. (i) Sugar transport and metabolism, sugars are secreted from plant roots via plant sugar transport proteins. Some microbes directly take up the monosaccharides or disaccharides [1], while others use plant invertases to hydrolyze disaccharides into a usable form [2]. (ii) Microbial preferences and catabolite repression, microbes prioritize specific sugars through carbon catabolite repression (CCR), selecting preferred energy sources while repressing the assimilation of others [3]. (iii) Sugar-mediated microbial behaviors, sugars act as chemoattractants, guiding bacterial movement towards the root environments [4]. They also influence microbial behaviors such as hyperflagellation [5] and biofilm formation [6], that enhance colonization. (iv) Symbiotic interactions, in mutualistic interactions, such as those with ectomycorrhizal fungi, sugars support fungal metabolism [7]. Fungi intracellularly transform these sugars into other forms (e.g., trehalose), that stimulate plant carbon allocation [8] and help sustain the symbiotic relationship. The transformed sugars, when released into the rhizosphere through damaged fungal cells, can attract the fungal-associated bacterial symbionts [9]. The figure was created in BioRender. Hemelda, NM (2025) https://BioRender.com/i98g972 (Accessed Jun 27, 2025).

Not surprisingly, the sequential use of carbon sources is governed by a hierarchical system, in which bacteria prioritize the consumption of the carbon source that supports the highest growth rate. In many cases, glucose is at the top of this hierarchy (Gralka et al. 2023; Wang et al. 2019). This hierarchical utilization reflects microbial preferences for specific carbon sources and is regulated by a mechanism known as catabolite repression (CCR). CCR suppresses the expression of genes and pathways involved in the metabolism of secondary carbon sources until the preferred source is depleted (Figure 1, [3]). This prioritization allows microbes to focus on carbon sources that provide the highest energy yield or are metabolically more efficient to process (Brückner and Titgemeyer 2002; Görke and Stülke 2008).

Hierarchical utilization of carbon sources is not only relevant for free-living microbes but also plays a crucial role in symbiotic relationships, where it influences the dynamics of carbon exchange between host plants and associated microorganisms. In the symbiotic interaction between plants and the ectomycorrhizal fungus *Laccaria bicolor*, the host plant secretes sucrose into the apoplastic space at the plant-fungus interface. This sucrose is hydrolyzed to glucose and fructose by plant invertases, with *L. bicolor* preferentially taking up glucose (Figure 1, [7]). The uptake of glucose triggers glucose-mediated CCR, which largely suppresses the assimilation of other carbon sources, such as fructose until the glucose supply is depleted (López et al. 2008). Once ingested, glucose is converted to trehalose, a fungus-specific carbon storage molecule that accumulates in fruiting body tissues, free-living mycelium, and particularly in the Hartig net hyphae (Deveau et al. 2008; López et al. 2007). This conversion not only ensures a steady demand for plant-derived sugars but also encourages the plant to continuously supply carbon to the mycorrhizal root, thereby maintaining the mutualistic relationship (Figure 1, [8]) (López et al. 2007).

In addition, trehalose accumulation in *L. bicolor* serves important ecological functions. Under nutrient-deficient or stressful conditions, trehalose can be released into the rhizosphere from decaying or damaged fungal hyphae. This release attracts beneficial bacteria such as *Pseudomonas fluorescens*, promoting a secondary mutualism that alleviates the stress effects on *L. bicolor* (Figure 1, [9]). This interaction highlights the dual role of trehalose—not only as a mediator of fungal symbiosis with plants but also as a key player in shaping microbial community dynamics within the rhizosphere (Deveau et al. 2010). While the mechanisms of carbon source preference and CCR have been well-studied in isolated contexts, their specific contributions to rhizosphere colonization, microbial adaptation, and plant-microbe interactions remain poorly understood and warrant further investigation.

## Sugars as communication signals

In plant-microbe interactions, sugars function both as essential nutrients and as key signaling molecules that facilitate complex communication between plants and their associated microbes. A prominent example is the role of sugars in modulating pathogen virulence. In *Xanthomonas campestris*, host-derived sucrose and glucose stimulate the expression of RpfF and RpfC, the diffusible signal factor (DSF) synthase and sensor, leading to DSF production that enhances bacterial pathogenicity (Zhang et al. 2019). In contrast, *Ralstonia pseudosolanacearum* exhibits a different sugar-mediated interaction involving arabinose. This root-exuded sugar cannot be metabolized by the pathogen; therefore, it has the potential to control bacterial wilt disease. In addition, exogenous application of arabinose reduces *R. pseudosolanacearum* populations in tomato stems and mitigates disease severity, likely through plant defense responses involving salicylic acid and ethylene, rather than antimicrobial effects or chemotaxis inhibition (Fu et al. 2020) (Table 1).

The relationship between plant immunity and root exudation further highlights the multiple roles of sugars. Following defoliation by mechanical injury or herbivory, concentrations of sucrose, glucose, and fructose in root exudates decline, which correlates with activation of the jasmonic acid (JA) signaling pathway. Concurrently, starch depletion occurs in damaged leaves, potentially altering carbon allocation to the roots (Castrillón-Arbeláez et al. 2012). These changes in sugar exudation, particularly the reduced fructose levels, may affect the microbial communities in the rhizosphere. For example, a JA-signaling *A. thaliana* mutant (*med25*) was shown to have elevated fructose levels in root exudates, which correlated with increased populations of rhizosphere microbes such as *Paenibacillus* spp., *Lysinibacillus* sp., and *Bacillus* sp. (Carvalhais et al. 2015).

Sugars also play a central role in beneficial plant-microbe interactions. In the interaction between maize and *Trichoderma virens*, the fungus uptakes sucrose exuded by the host plant. Unlike fungi that depend on plant invertases for sucrose hydrolysis (e.g., *Laccaria bicolor*, as discussed in Sugars as carbon sources for microbes), *T. virens* hydrolyzes sucrose to glucose and fructose intracellularly using its own invertase (TvInv). This independent sucrose utilization supports fungal metabolism and influences its interaction with maize by enhancing carbon allocation to the roots. The increased sucrose breakdown by *T. virens* has been linked to higher photosynthetic rates of the host plant, suggesting a coordinated exchange of resources that supports the sustainability of this mutualistic relationship (Vargas et al. 2009). Another example of a sugar-mediated interaction is that *Trichoderma atroviride* modulates sucrose exudation in tomato roots, as sucrose is detected in root exudates only in the presence of this fungus. Although the purpose and mechanisms of this modulation remain unclear, sucrose promotes fungal growth in vitro similarly to other sugars such as xylose, glucose, *myo*-inositol, and fructose. These examples illustrate the diverse signaling roles of sugars in shaping plant-microbe interactions, ranging from enhancing pathogen virulence to mediating plant immunity and maintaining beneficial symbioses.

## Sugars as facilitators of root colonization

Sugars in root exudates play a pivotal role in driving microbial colonization within the rhizosphere (Chaparro et al. 2013; Mesny et al. 2023) (Table 1). In maize, simpler sugars such as sucrose and monosaccharides (e.g., glucose and mannose) exert a significant influence on the root microbiome, particularly during early developmental stages, while more complex sugars such as trehalose, arabinose, and galactose become increasingly influential during later stages of plant development. Early-stage plants exude higher amounts of sucrose and monosaccharides, which serve as common carbon sources and chemoattractants, promoting the colonization of generalist microbes (Bhattacharjee et al. 2012; Deveau et al. 2008; Feng et al. 2022; Vargas et al. 2009) (Figure 1, [4]). As root microbiomes become established, plants refine microbial communities by selectively promoting beneficial microbes to enhance adaptation to environmental stress, a process mediated by the exudation of complex sugars such as trehalose. Trehalose is particularly important for symbiotic microorganisms, including AMF and rhizobia, as well as non-symbiotic root-associated microbes. This sugar enhances microbial survival under stress conditions, such as drought and salinity, which eventually contribute to improving the stress tolerance of host plants (Onwe et al. 2022; Sharma et al. 2020).

The mechanisms by which sugars influence root colonization continue to be the subject of active investigation. While their role as carbon sources providing energy to fuel microbial growth is fundamental to promoting colonization (Deveau et al. 2008; O’Banion et al. 2023), emerging evidence indicates that sugars play a much more dynamic role. Beyond their metabolic function, sugars significantly modulate microbial behaviors such as chemotaxis and biofilm formation—key traits essential for successful root colonization (Cole et al. 2017). Sugars can act as chemoattractants that promote microbial motility toward plant roots (Bhattacharjee et al. 2012; Feng et al. 2018, 2022; Liu et al. 2020; Tian et al. 2021) (Table 1). For example, glucose released from rice strongly attracts *Rhizobium leguminosarum* bv. *trifolii*, thereby enhancing root colonization. Similarly, monosaccharides (e.g., fructose, mannose, fucose, galactose, ribose), disaccharides (e.g., sucrose, maltose), and sugar alcohols (e.g., dulcitol, ribitol) act as chemoattractants for various *Bacillus* species (Feng et al. 2018, 2022; Liu et al. 2020; Tian et al. 2021). These sugars are recognized by different chemoreceptors. In *Bacillus amyloliquefaciens*, maltose, fructose, and dulcitol are sensed via the methyl-accepting chemotaxis protein (MCP) TlpB, whereas mannose, fucose, ribose, and ribitol bind directly to the McpA chemoreceptor (Feng et al. 2018, 2022). Interestingly, D-galactose is also recognized by McpA in *B. velezensis*, but through an indirect mechanism, possibly mediated by a galactose-binding protein (Liu et al. 2020). In *B. subtilis*, sucrose-induced motility involves hyper-flagellation and increased surfactin production, which is regulated by the *srf* operon. Notably, sucrose alone, but not its hexose derivatives (e.g., glucose and fructose), is able to induce this operon and increase surfactin production (Tian et al. 2021) (Figure 1, [1], [5], [6]).

## Conclusion and future perspectives

Sugars are crucial components of plant-microbe interactions in the rhizosphere, serving as carbon sources and signaling molecules that influence microbial behavior and community composition. Although progress has been made, significant gaps remain in our understanding of these processes. An ongoing challenge is to link genomic and metabolomic data to better understand the genetic basis of sugar diversity in root exudates. Although variations in sugar profiles across species and conditions have been documented, the genetic mechanisms underlying these differences have not been fully elucidated. Studies such as those by Mönchgesang et al. (2016) and Subrahmaniam et al. (2025) provide initial insights by connecting specific metabolites, including sugars, to genetic loci or candidate genes, although most focus on broader classes of metabolites rather than sugars specifically. The combination of genomic tools with metabolomic analyses may provide further opportunities to explore the regulatory pathways and genetic factors involved in sugar exudation. Another important challenge is the accurate measurement of labile sugars such as glucose, which are rapidly consumed by microbes in the rhizosphere. The high turnover rate of these sugars, often within minutes of exudation, complicates their detection and quantification, especially in natural soil systems. Methodological advances and the development of in situ analytical tools will be crucial to overcome these limitations and improve our understanding of the role of labile sugars in plant-microbe interactions. In addition, certain aspects of sugar-mediated interactions remain underexplored. For instance, while the roles of glucose, sucrose, and fructose have been well-studied, other sugars such as arabinose and trehalose have received less attention. These sugars show potential in shaping microbial interactions and promoting plant resilience under stress conditions, but their mechanisms and ecological implications require further investigation. Similarly, microbial sugar transport mechanisms are less understood compared to plant sugar transport systems, particularly the role of microbial transporters in sensing and utilizing exuded sugars. Thus, investigations of understudied sugars and microbial sugar transporters could provide new insights into the complexity of rhizosphere processes. These efforts, while incremental, hold potential to improve our understanding of sugar-mediated interactions and their application to sustainable agricultural practices.

## Abbreviations

AMF: arbuscular mycorrhizal fungi
CCR: carbon catabolite repression
DSF: diffusible signal factor
JA: jasmonic acid
MCP: methyl-accepting chemotaxis protein
P: phosphorus
